# Methylation silencing CDH23 is a poor prognostic marker in diffuse large B-cell lymphoma

**DOI:** 10.18632/aging.203268

**Published:** 2021-07-12

**Authors:** Baoping Cao, Xiaochuan Guo, Lefu Huang, Bin Wang, Weixia Wang, Dong Han, Weijing Zhang, Kaili Zhong

**Affiliations:** 1 Department of Lymphoma, Beijing Shijitan Hospital, Capital Medical University, Haidian 100038, Beijing, China

**Keywords:** cadherin-23, diffuse large B-cell lymphoma, DNA methylation, CDK1, CDK2

## Abstract

Cadherin-23(CDH23) mediates homotypic and heterotypic cell-cell adhesions in cancer cells. However, the epigenetic regulation, the biological functions, the mechanisms and the prognostic value of CDH23 in diffuse large B-cell lymphoma (DLBCL) are still unclear. The Gene Expression Profiling Interactive Analysis (GEPIA) and the Gene Expression Omnibus (GEO) database were employed to analyze the CDH23 expression level in DLBCL. The correlation of CDH23 expression and methylation was analyzed by LinkedOmics database. The prognostic value was analyzed via GEPIA. Correlated genes, target kinase, target miRNA, target transcription factor and biological functions were identified by LinkedOmics and GeneMANIA database. The relationship between CDH23 and the immune cell infiltration was explored by the Tumor Immune Estimation Resource (TIMER). The expression of CDH23 was reduced by DNA methylation significantly in DLBCL tissue. Reduction of CDH23 represented poor outcome of DLBCL patients. Functional enrichment analysis showed that CDH23 mainly enriched in cancer cell growth, cell metastasis, cell adhesion, cell cycle, drug catabolic process, leukocyte mediated immunity and DNA repair by some cancer related kinases, miRNAs and transcription factors. These results indicated that methylated reduction of CDH23 represented poor outcome of DLBCL. CDH23 is associated with essential biological functions and key molecules in DLBCL. CDH23 may play crucial roles in DLBCL tumorigenesis. Our results lay a foundation for further investigation of the role of CDH23 in DLBCL tumorigenesis.

## INTRODUCTION

Diffuse large B-cell lymphoma (DLBCL), which is characterized by clinical and biological heterogeneity, is a major subtype of non-Hodgkin’s lymphoma [1]. Although more than 50% of DLBCL patients can achieve durable remission, approximate one-third of cases cannot be cured by standard-of-care immunochemotherapeutic regimens, which is still a challenging clinical problem [2, 3]. Currently, limitations of effective treatment are associated with the heterogeneity of DLBCL partly, including the clinical level, immunophenotypic, morphologic and genetic heterogeneity [4]. The high-risk DLBCL patients may achieve more effective treatment and have better outcome, if they can be identified more prospectively. Genome-wide molecular analysis of DLBCL revealed abundant of altered cellular pathways, which play important roles in development, maintenance and response to therapy of tumor [4, 5]. Growing evidences demonstrate that epigenetic aberrations play important roles in carcinogenesis and tumor progression. DNA methylation often occurs in multiple cancer-related signaling pathways, which is usually involved in cell cycle, DNA damage repair, Wnt signaling, TGF-β signaling and NF-κB signaling [6–10]. Epigenetic programming aberration like DNA methylation, has also emerged as a hallmark in multiple hematological malignancies including DLBCL [11, 12].

Cadherin is a large family of transmembrane glycoproteins. Cadherin takes part in embryogenesis, cell proliferation, tissue architecture, and signal transductions in multicellular organizations. Cadherin mediates cell-cell adhesion depending Ca^2+^ [13–16]. Cadherin-23 (CDH23), with a long EC region remarkably, is a non-classical cadherin, and comprises calcium- depending cell-cell adhesion glycoproteins [17]. Similar to classical cadherins, the localization of CDH23 at the cell-cell junction was showed in breast cancer cell MCF7 *in vitro*. CDH23 was identified at the contacts between normal breast fibroblasts and MCF7, and mediated homotypic and heterotypic cell-cell adhesions between fibroblasts and epithelial cells *in vitro* [18]. CDH23 expression was also identified at the cell boundaries in various normal mice tissues, including muscle, kidney, brain, heart and testes. CDH23 proteins were also expressed in esophageal squamous cell carcinoma (ESCC) and human lung cancer (LC) at the cell boundaries in both cancer and normal tissues. CDH23 was downregulated through promoter methylation in LC and ESCC cells [19]. CDH23 can suppress cancer cell migration and promote aggregation *in vitro*. Case studies with tumor patients suggested that the expression level of CDH23 associated with patient survival and metastasis [19]. However, the epigenetic regulation, the biological functions, the mechanisms and the prognostic value of CDH23 in DLBCL are still unclear. In this study, we investigated the epigenetic regulation, the expression level and prognostic value of CDH23 in DLBCL, and further analyzed the potential biological functions of CDH23 in DLBCL by comprehensive bioinformatic analysis.

## RESULTS

### CDH23 expression was reduced in DLBCL via DNA methylation and reduction of CDH23 was correlated with poor outcome of DLBCL patients

**Figure 1:**
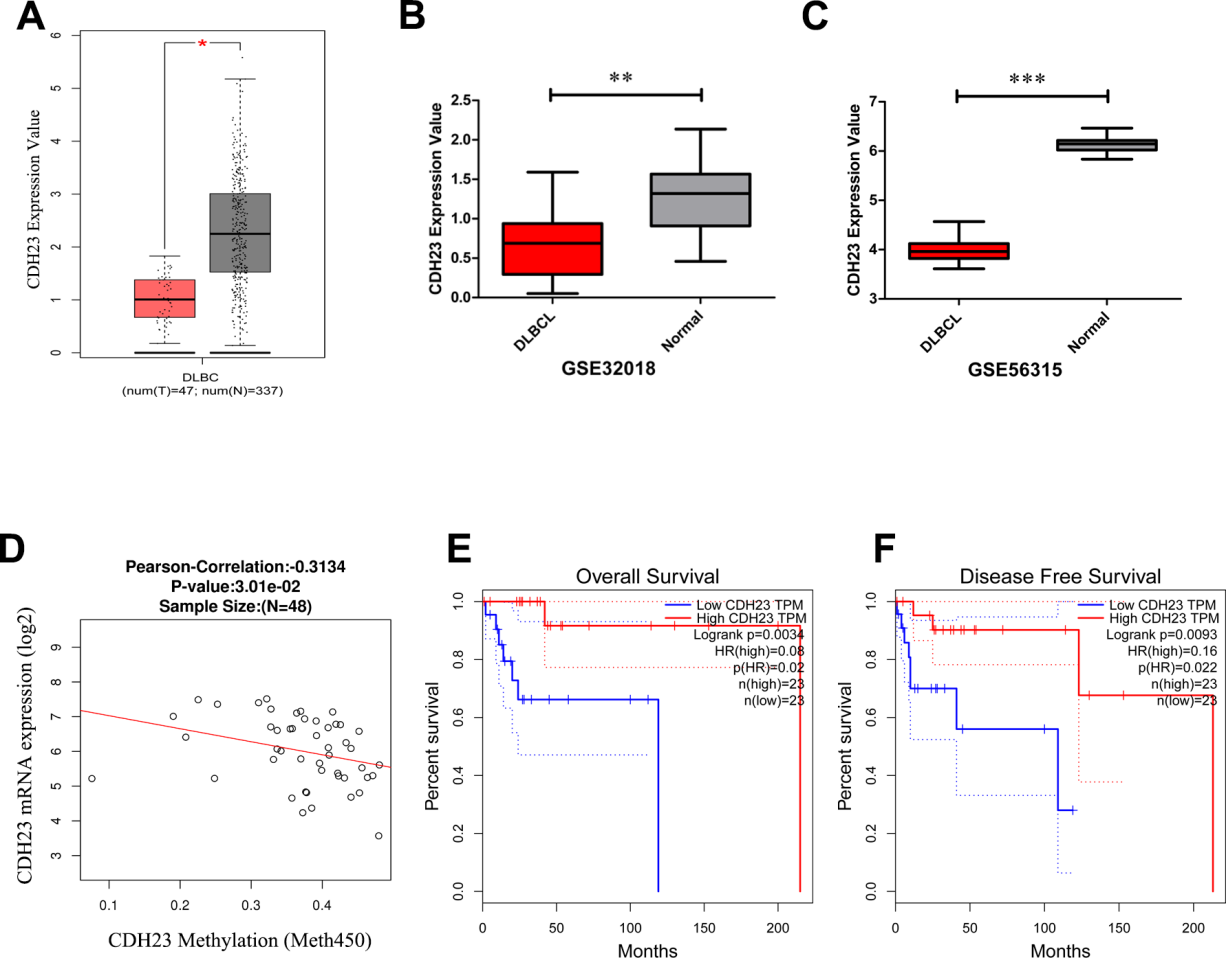
**The expression level, epigenetic regulation and prognostic value of CDH23 in DLBCL.** (**A**) The expression level of CDH23 in DLBCL samples and paired normal samples determined by GEPIA. (**B**) The expression level of CDH23 in DLBCL samples and paired normal samples in GSE32018 dataset. (**C**) The expression level of CDH23 in DLBCL samples and paired normal samples in GSE56315 dataset. (**D**) The correlation of CDH23 expression and methylation value in DLBCL via LinkedOmics analysis. (**E**) The relationship between CDH23 expression and overall survival of DLBCL patients via GEPIA analysis. (**F**) The relationship between CDH23 expression and disease free survival of DLBCL patients via GEPIA analysis.

To explore the expression of CDH23 in DLBCL and corresponding normal samples, the GEPIA database and GEO database were employed. The expression level of CDH23 was significantly reduced in DLBCL tissues than normal tissues in GEPIA database (Figure 1A, *p*<0.05), GSE32018 dataset from GEO database (Figure 1B, *p*<0.01) and GSE56315 dataset from GEO database (Figure 1C, *p*<0.0001). The methylation value of *CDH23* and expression level of CDH23 was correlated negatively via LinkedOmics analysis (Figure 1D, cor=-0.3134, *p*<0.05). The expression level of CDH23 was upregulated after the treatment of demethylating agent decitabine for 48 hours in DLBCL cell line Su-DHL6, OCI-Ly10 and OCI-Ly1 (Supplementary Figure 1D). These results suggested that the expression of CDH23 was significantly downregulated in DLBCL via DNA methylation. We verified these results in breast invasive carcinoma (BRCA) via the DNA methylation interactive visualization database (DNMIVD) analysis. The CDH23 expression was significantly reduced in BRCA tissues than normal tissues (*p*=2.77e-59, Supplementary Figure 1A). *CDH23* was hyper-methylated in BRCA tissues than normal tissues (*p*=1.83e-03, Supplementary Figure 1B). The expression value of CDH23 and promoter methylation value was correlated negatively in BRCA (cor=-0.16, *p*=4.51e-06, Supplementary Figure 1C). Further we explored the clinical value of CDH23 for the prognosis of DLBCL. The survival analysis formed by GEPIA database indicated that reduction of CDH23 represented poor overall survival (OS) (Figure 1E, *p*<0.01), as well as poor disease-free survival (DFS) in DLBCL patients (Figure 1F, *p*<0.01). These results indicated that methylation of *CDH23* could be a reliable prognostic factor in DLBCL.

### Genetic alterations of CDH23 in DLBCL

**Figure 2:**
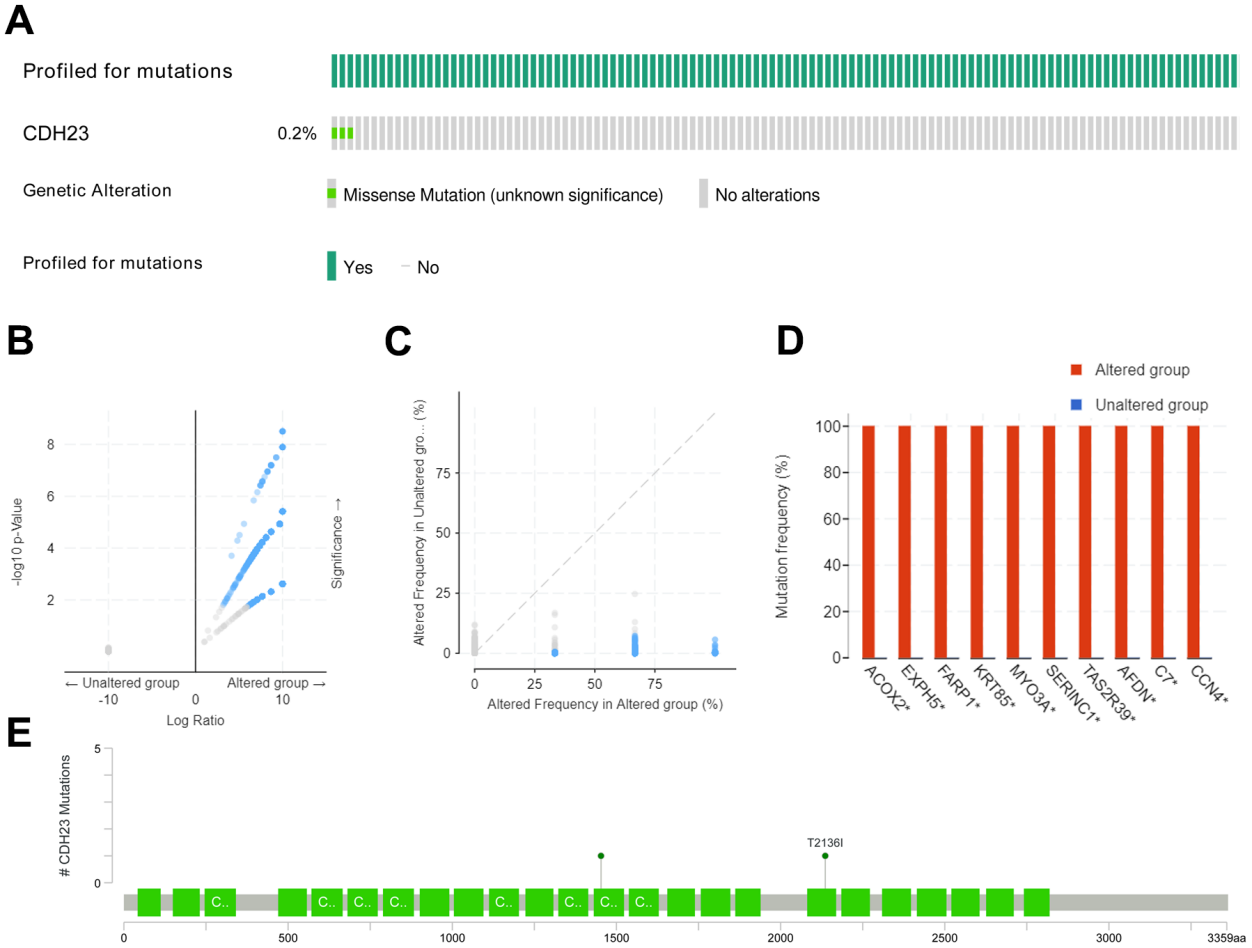
**Mutated-types, co-mutations and the mutation location of *CDH23* in DLBCL.** (**A**) Aberration types and frequency of *CDH23* in DLBCL. (**B**) Volcano plots of mutations in *CDH23* altered group and unaltered group. Blue dots denote genes mutated significantly (p<0.05), and grey dots denote genes don’t mutate significantly (p≥0.05). (**C**) Aberration frequency scatter in *CDH23* altered group and unaltered group. Blue dots denote genes mutated significantly (p<0.05), and grey dots denote genes don’t mutate significantly (p≥0.05). (**D**) Co-mutations with *CDH23* in DLBCL. (**E**) Locations of *CDH23* mutations in DLBCL. CNA: DNA copy-number alteration.

Different mutated types, such as missense, amplification, deep deletion and so on, may play distinct roles in gene functions. The mutated-types, co-mutations and the mutated location of *CDH23* were analyzed through c-BioPortal web analysis tool. The results showed that there was a missense mutation of *CDH23* in 0.2% DLBCL tissues (Figure 2A). The genetic altered frequency in the *CDH23* altered group was higher than that in the *CDH23* unaltered group (Figure 2B, 2C). The most frequent co-mutated genes of *CDH23*, including *ACOX2*, *EXPH5*, *FARP1*, *KRT85*, *MYO3A*, *SERINC1*, *TAS2R39*, *AFDN*, *C7* and *CCN4* were analyzed in the *CDH23* altered group and unaltered group too. And these co-mutated genes mutated more frequently in the *CDH23* altered group than the unaltered group (Figure 2D). Missense mutation of *CDH23* existed in 3 DLBCL samples, the protein change of CDH23 in two of the samples was T2136I, and A1453V in the other one (Figure 2E).

### Correlated significant gene analysis of CDH23 in DLBCL

**Figure 3:**
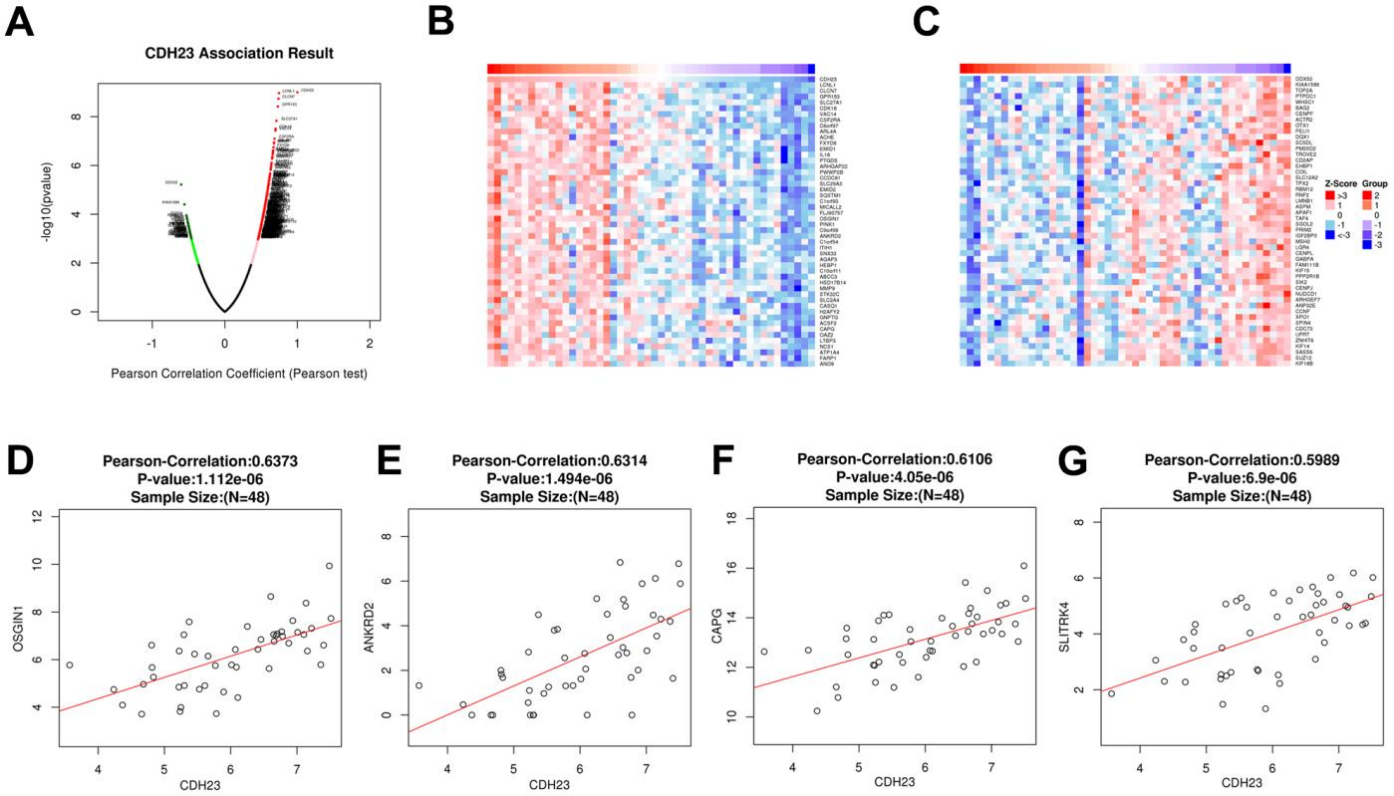
**Correlated significant genes of CDH23 and gene correlation expression analysis for CDH23 in DLBCL (LinkedOmics).** (**A**) Volcano plots of CDH23 correlated genes in DLBCL. Red suggests positively correlated genes and green shows negatively correlated genes. (**B**) Heat map of positively correlated genes with CDH23 in DLBCL, respectively (top 50). (**C**) Heat map of negatively correlated genes with CDH23 in DLBCL, respectively (top 50). (**D**) The scatter plots shows Pearson-correlation of CDH23 expression with OSGIN1. (**E**) The scatter plots shows Pearson-correlation of CDH23 expression with ANKRD2. (**F**) The scatter plots shows Pearson-correlation of CDH23 expression with CAPG. (**G**) The scatter plots shows Pearson-correlation of CDH23 expression with SLITRK4. OSGIN1, oxidative stress induced growth inhibitor 1. ANKRD2, ankyrin repeat domain 2. CAPG, capping actin protein, gelsolin like. SLITRK4, SLIT and NTRK like family member 4.

**Figure 4:**
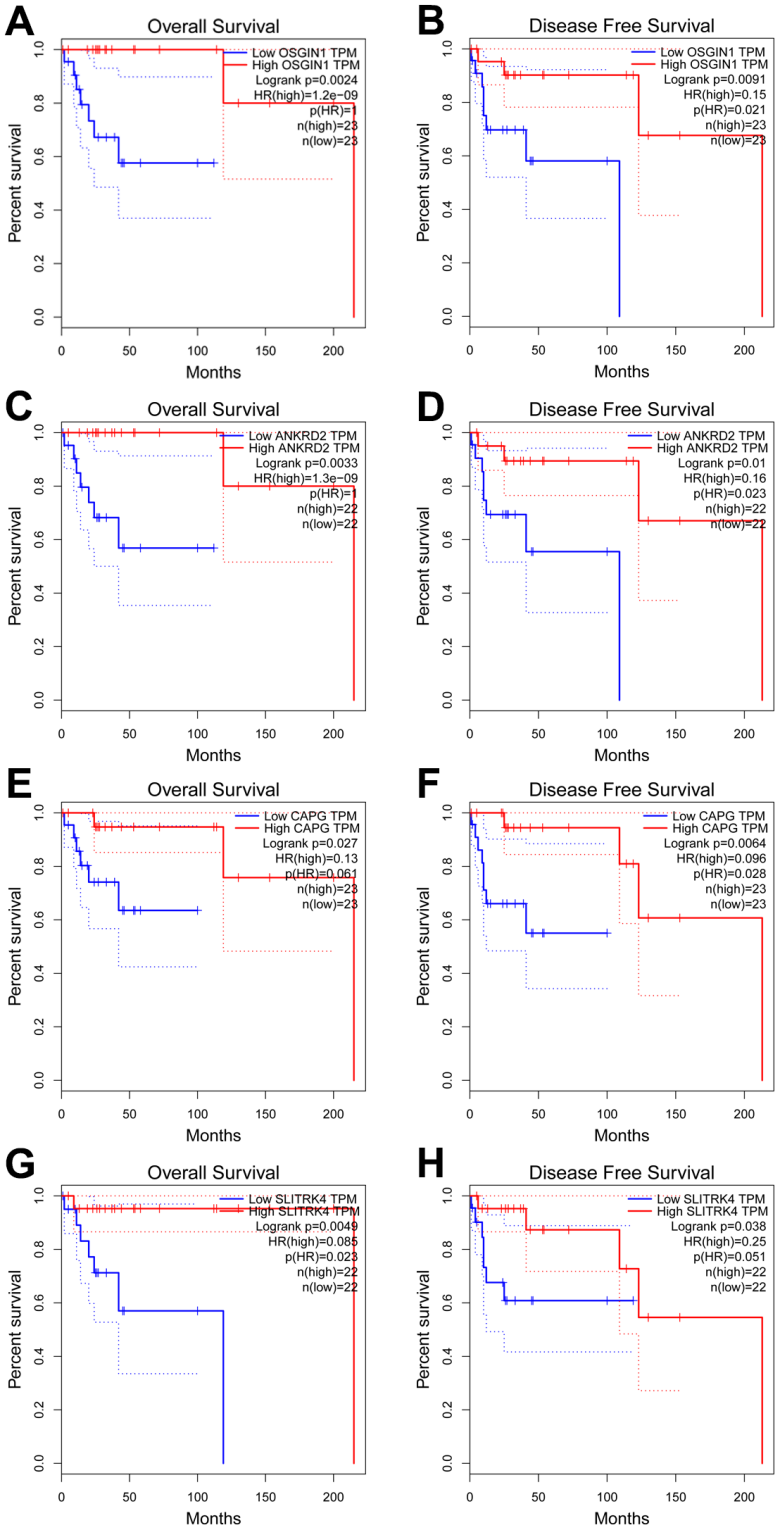
**Prognostic analysis of genes correlated with CDH23 in DLBCL (GEPIA).** (**A**, **B**) The relationship between OSGIN1 expression and overall survival and disease free survival of DLBCL patients. (**C**, **D**) The relationship between ANKRD2 expression and overall survival and disease free survival of DLBCL patients. (**E**, **F**) The relationship between CAPG expression and overall survival and disease free survival of DLBCL patients. (**G**, **H**) The relationship between SLITRK4 expression and overall survival and disease free survival of DLBCL patients.

To explore the potential function and mechanism of CDH23 in DLBCL, correlation analysis between CDH23 and various genes was performed via LinkedOmics. The top 50 positively and negatively significantly correlated genes were showed in Figure 3A–3C. CDH23 expression was positively interacted with LCNL1, CLCN7, GPR153, SLC27A1, CDK18, etc. Otherwise CDH23 expression was negatively related with DDX52, TOP2A, PTPDC1, etc. Further we selected the significantly correlated genes (cor≥0.5) of CDH23 to conduct prognosis analysis in DLBCL via GEPIA database. There were four significantly correlated genes of CDH23, including OSSGIN1 (Pearson correlation=0.6373, *p*=1.112e-06), ANKRD2 (Pearson correlation=0.6314, *p*=1.494e-06), CAPG (Pearson correlation=0.6106, *p*=4.05e-06) and SLITRK4 (Pearson correlation=0.5989, *p*=6.9e-06) consistent with selected threshold (Figure 3D–3G). The prognostic value of the selected genes in DLBCL was further analyzed via GEPIA database. The results suggested that reduction of OSGIN1 expression represented poor overall survival (OS) (Figure 4A, *p*=0.0024), as well as poor disease-free survival (DFS) in DLBCL patients (Figure 4B, *p*=0.0091). And reduction of ANKRD2, CAPG and SLITRK4 also represented poor OS, as well as poor DFS in DLBCL patients (Figure 4C–4H).

### The relationship between CDH23 and immune cell infiltration via TIMER analysis

**Figure 5:**
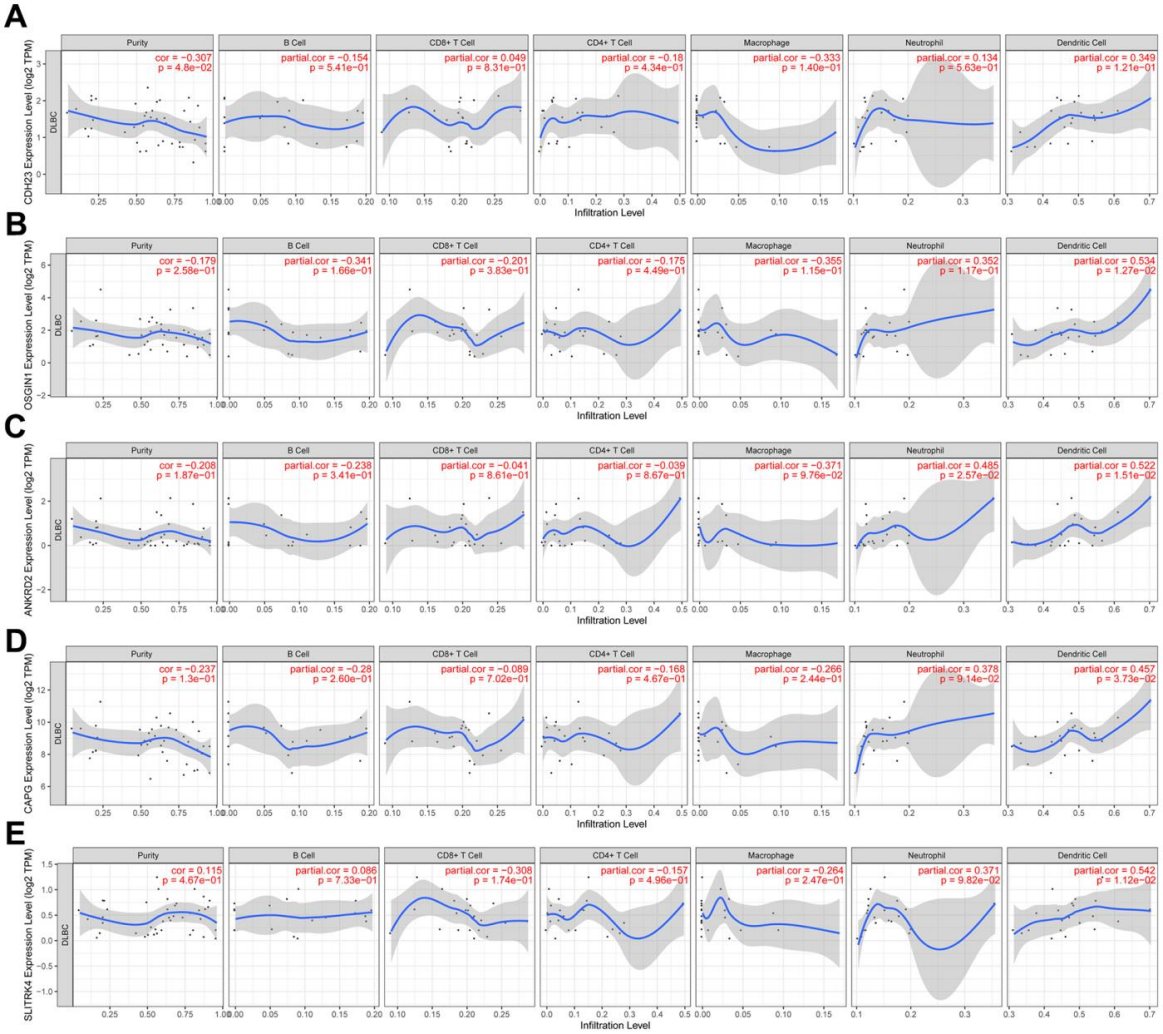
**The relation between expression of CDH23 and CDH23 related genes and immune cells infiltration in DLBCL (TIMER).** (**A**) Association between CDH23 and several types of immune cell infiltration. (**B**) Association between OSGIN1 and several types of immune cell infiltration. (**C**) Association between ANKRD2 and several types of immune cell infiltration. (**D**) Association between CAPG and several types of immune cell infiltration. (**E**) Association between SLITRK4 and several types of immune cell infiltration.

Recently, because of the clinical successes of immunotherapy for cancer, the investigation of the interaction between malignant cells and host immune system is necessitated. The immune cell infiltration has an important influence on the prognosis of some cancer types. The relationship between CDH23 expression and immune cell infiltration was investigated in this study via TIMER database analysis. The results showed that CDH23 had a negatively significant correlation with tumor purity (Figure 5A, cor=-0.307, *p*=4.8e-02). Further we explored the relationship between CDH23 related genes OSGIN1, ANKRD2, CAPG, SLITRK4 and immune cell infiltration. The results suggested that OSGIN1 had a positively significant correlation with dendritic cell infiltration (Figure 5B, cor=0.534, *p*=1.27e-02). ANKRD2 had a positively significant correlation with neutrophil cell infiltration (cor=0.485, *p*=2.57e-02) and dendritic cell infiltration (cor=0.522, *p*=1.51e-02) as showed in Figure 5C. Both CAPG (cor=0.457, *p*=3.73e-02) and SLITRK4 (cor=0.542, *p*=1.12e-02) had a positively significant correlation with dendritic cell infiltration (Figure 5D, 5E).

### Enrichment function analysis of CDH23

**Figure 6:**
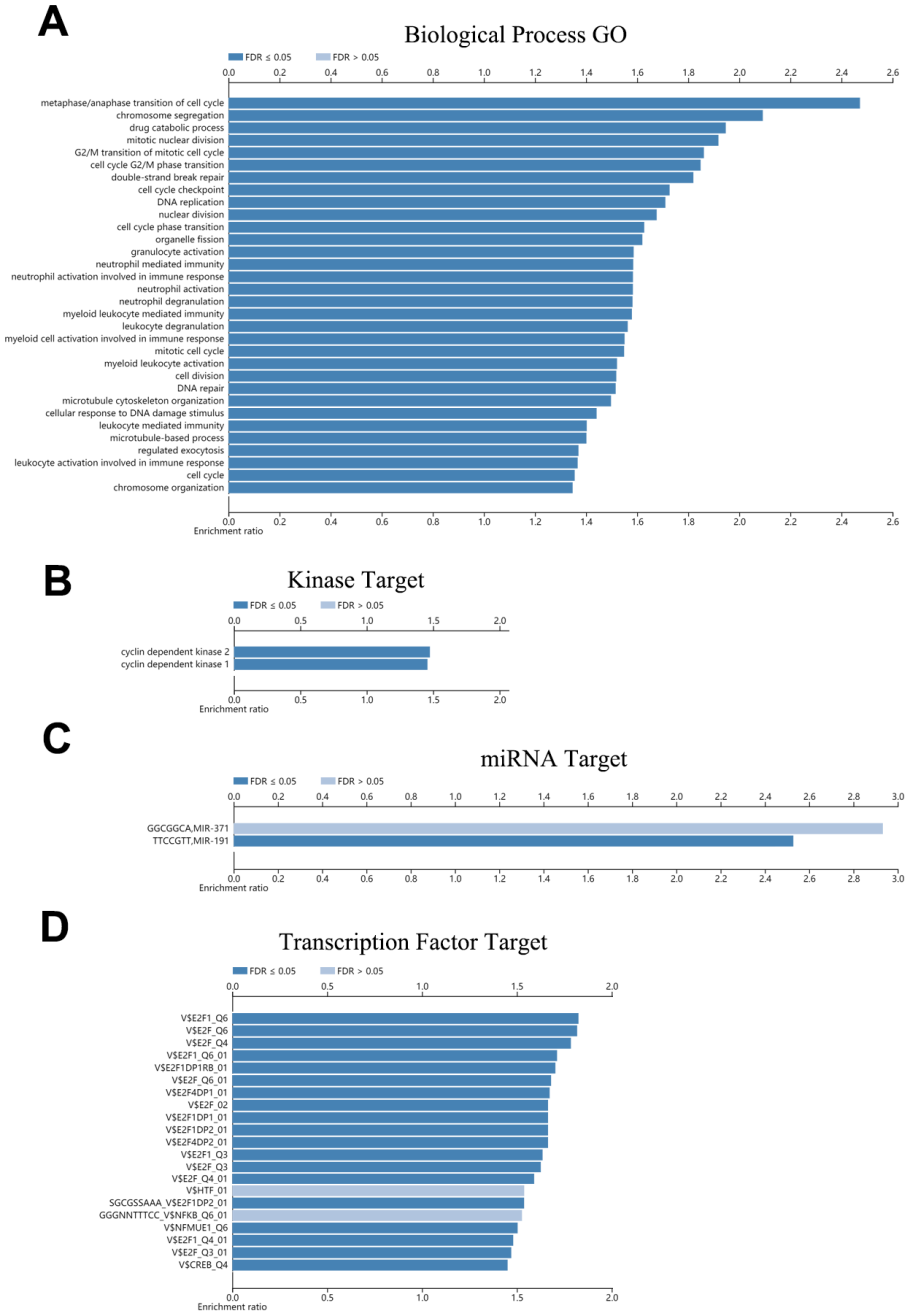
**Function enrichment, kinases targets, miRNA targets and transcription factor targets of CDH23 (LinkedOmics).** (**A**) Biological process enrichment of CDH23 in DLBCL. (**B**). Kinase targets of CDH23 in DLBCL. (**C**) MiRNA target of CDH23. (**D**) Transcription factor targets of CDH23.

To investigate the potential biological functions of CDH23, we used the LinkOmics database to analyze the potential biological process of CDH23 in DLBCL (Figure 6A, Supplementary Table 1). The results indicated that CDH23 associated with cell cycle process, drug catabolic process, leukocyte mediated immunity, regulated exocytosis, DNA replication, nuclear division, DNA repair, chromosome organization, and so on. These results indicated that CDH23 may play important roles in some crucial biological process and molecular functions, which suggested that CDH23 played a key role in DLBCL progression. To explore the significant kinases, miRNA targets and transcription factor targets of CDH23 in DLBCL, the LinkedOmics database was employed in this study. The significant kinase targets of CDH23 included cyclin dependent kinase 1 (CDK1) and cyclin dependent kinase 2 (CDK2) (Figure 6B, Supplementary Table 2). The significant miRNA target of CDH23 was (TTCCGTT) MIR-191 (Figure 6C, Supplementary Table 3). The significant transcription factor targets of CDH23 included E2F1, E2F4, E2F1DP2, and so on (Figure 6D, Supplementary Table 4). These results further showed that CDH23 played important roles in DLBCL via interacting with CDK1, CDK2, E2F1, E2F4, E2F1DP2 and MIR-191.

Further we found CDK1 was upregulated in DLBCL tissues (p<0.05, Supplementary Figure 2A), and the expression level of CDK1 and CDH23 associated negatively (cor=-0.316, *p*=2.88e-02, Supplementary Figure 2B). CCNB1 was upregulated in DLBCL tissues (p<0.05, Supplementary Figure 2C), and the expression level of CCNB1 and CDH23 associated negatively as well (cor=-0.295, *p*=4.15e-02, Supplementary Figure 2D). These results verified the function of CDH23 in regulating of cell cycle in DLBCL.

### PPI network analysis of CDH23 via GeneMANIA

**Figure 7:**
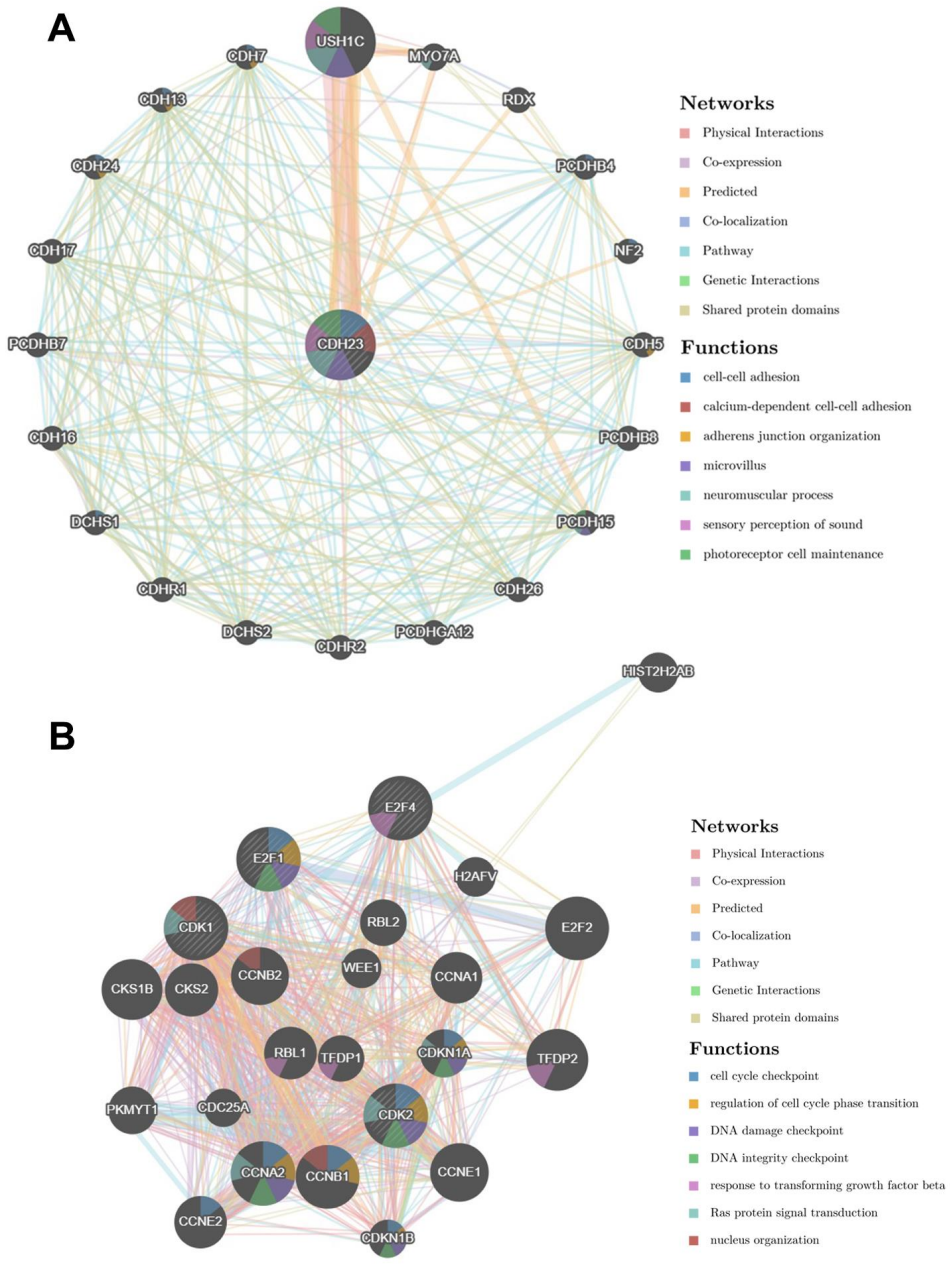
**Protein-protein interaction network of CDH23 and its target proteins (GeneMANIA).** (**A**) Protein-protein interaction network of CDH23. The gene set enriched in the target network of CDH23 was explored via Protein-protein interaction (PPI) network and functional analysis. Distinct colors of the network edge indicate the bioinformatic methods applied: physical interactions, co-expression, predicted, co-localization, pathway, genetic interactions and shared protein domains. The distinct colors for the network nodes show the biological functions of the sets of enrichment genes, including cell-cell adhesion, adherens junction organization, microvillus, neuromuscular process, sensory perception of sound and photoreceptor cell maintenance. (**B**) Protein-protein interaction network of CDH23 target proteins, including CDK1, CDK2, E2F1 and E2F4. Distinct colors of the network edge indicate the bioinformatic methods applied: physical interactions, co-expression, predicted, co-localization, pathway, genetic interactions and shared protein domains. The distinct colors for the network nodes show the biological functions of the sets of enrichment genes, including cell cycle checkpoint, regulation of cell cycle phase transition, DNA damage checkpoint, DNA integrity checkpoint, response to transforming growth factor beta, Ras protein signal transduction and nucleus organization.

The PPI network was constructed via GeneMANIA to analyze the CDH23 interaction with other genes. The PPI network showed that CDH23 significantly interacted with USH1C, MYO7A, RDX, PCDHB4, NF2, CDH5 and other essential genes. The biological functions of these genes may associate with deafness, cancer cell growth, cell metastasis, cell adhesion, and so on (Figure 7A). These results indicated that CDH23 takes crucial part in cancer progress. Further the PPI network of CDH23 targeting proteins CDK1, CDK2, E2F1 and E2F4 was analyzed, which indicated that CDH23 may regulate cell cycle, DNA damage, TGFβ signaling and Ras protein signaling via these target proteins (Figure 7B).

## DISCUSSION

Numeral investigations have tried to explore the pathogenesis and potential mechanisms of DLBCL, which have provided chances for the diagnosis and treatment of DLBCL. Recently, integrated bioinformatic analysis has been used progressively to explore cancer pathogenesis, development of potential biomarkers for diagnosis, prognostic biomarkers and therapeutic molecular targets. For example, CCL18, a single molecular biomarker, was identified via bioinformatics analysis for the diagnosis and treatment of DLBCL [20]. It was reported that CDH23 was downregulated via DNA methylation in various tumors, and suppressed cancer cell migration and promoted aggregation of cancer cells. CDH23 expression was related with survival and metastasis of patients [19]. Epigenetic alteration of *CDH23* may play important roles in cancer progression. The function and mechanism of CDH23 in DLBCL need further investigation.

The expression level of CDH23 between normal and DLBCL samples was analyzed via bioinformatic analysis. The expression level of CDH23 was lower in DLBCL samples than corresponding normal samples. CDH23 expression was related negatively with methylation value of *CDH23*. The expression level of CDH23 was upregulated after the treatment of demethylating agent decitabine in DLBCL cell lines. Reduction of CDH23 expression represented poor overall survival, as well as poor disease-free survival in DLBCL patients. These results indicated that the expression of CDH23 was regulated by DNA methylation. The methylation of *CDH23* may serve as a detective and prognostic biomarker of DLBCL. Further the genetic alterations results of *CDH23* in DLBCL showed that there was a missense mutation of *CDH23* in 0.2% DLBCL samples, and the genetic altered frequency in the *CDH23* altered group was higher than that in the *CDH23* unaltered group. These results suggested that the genetic alteration of *CDH23* may be an important factor of whole genomic stability. The most frequent co-mutated genes of *CDH23* included *ACOX2*, *EXPH5*, *FARP1*, *KRT85*, *MYO3A*, *SERINC1*, *TAS2R39*, *AFDN*, *C7* and *CCN4*. It was reported that variants of *ACOX2* was associated with cardiovascular disease and breast cancer, and may serve as a cancer metabolism hallmark [21–23]. Overexpression of FARP1 was significantly correlated with lymph metastasis, lymphatic invasion and poor prognosis in advanced gastric cancer patients. FARP1 promoted cell motility through activating CDC42 in gastric cancer [24]. MYO3A was reported to serve as a prognostic marker for tracking progression of breast cancer toward metastasis [25]. These studies indicated that the mutation of *CDH23* may play crucial roles in DLBCL progression.

Correlation analysis was taken between CDH23 and various genes. CDH23 expression was positively associated with LCNL1, CLCN7, GPR153, SLC27A1, CDK18, etc. CDH23 expression was negatively related with DDX52, KIAA1586, TOP2A, PTPDC1, WHSC1, and so on. There were four significantly correlated genes of CDH23, including OSSGIN1, ANKRD2, CAPG and SLITRK4, reduction of which represented poor overall survival (OS), as well as poor disease-free survival (DFS) in DLBCL patients. These results indicated the important prognostic value of CDH23 and its correlated genes in DLBCL patients.

In tumorigenesis and progression, the cancer microenvironment also has a vital role. One of the most critical elements of the cancer microenvironment is the immune cell infiltration. Recently, advances in immunotherapy, especially checkpoint blockade, have resulted in clinical success in treatment of late-stage cancers [26]. As for unsatisfactory outcomes for those relapsed and refractory DLBCL patients, it needs more efforts to discover new therapy approaches, such as immunomodulatory drugs, immune checkpoint inhibitors, and so on [27]. The relationship analysis between CDH23 expression and immune cell infiltration showed that CDH23 had a negatively significant correlation with tumor purity. The CDH23 related genes OSSGIN1, ANKRD2, CAPG and SLITRK4 correlated with dendritic cell infiltration and neutrophil cell infiltration. Dendritic cell can induce immune memory response in cancer and promote anti-tumor immunity. CDH23 may play important role in microenvironment of DLBCL.

The PPI network and the enrichment functions of CDH23 showed that CDH23 took part in the essential biological functions of DLBCL, including cancer cell growth, cell metastasis, cell adhesion, cell cycle, drug catabolic process, leukocyte mediated immunity, DNA replication, nuclear division, DNA repair, chromosome organization, and so on. Among these biological processes, cancer cell growth, cell metastasis, cell adhesion, cell cycle, DNA repair, and immunity response play critical roles in lymphoma progression. CDH23 was also associated with drug catabolic process, which suggested that CDH23 may serve as a biomarker of chemosensitivity of DLBCL. PPI network of CDH23 targeting proteins CDK1, CDK2, E2F1 and E2F4 was analyzed as well, which indicated that CDH23 may regulate cell cycle, DNA damage, TGFβsignaling, Ras protein signaling, and so on, via interaction with these target proteins.

Cyclin-dependent kinases (CDKs), a specific family of serin/threonine kinases, plays an essential role in cell cycle regulation, that allows transition between its different phase [28]. CDKs includes a mitotic CDK (CDK1), three interphase CDKs (CDK2, CDK4, and CDK6), a regulatory CDK (CDK7), and transcriptional CDKs (CDK8 and CDK9) [29]. Cyclin A, which accumulates at the G1/S phase boundary, can activate CDK2 and CDK1, and then promotes progression through the G2 interval. B-type cyclins, especially CCNB1, interacting with CDK1 drive cells into mitosis at this point [28]. Alterations of CDK activity mediate tumor-associated cell cycle defects frequently. Misregulation of CDKs often induces unscheduled proliferation, genomic instability and chromosomal instability as well [30]. Several investigations reported that CDK1 was upregulated in various malignancies. CDK1 was also reported to be associated with chemotherapy resistance in tumors [31, 32]. The alteration of CDK2 was also found in several cancers, such as glioblastoma, B cell lymphoma and so on [33, 34]. In this study, the significantly kinase targets of CDH23 included CDK1 and CDK2, which indicated that CDH23 may play key roles in DLBCL via interaction with CDK1 and CDK2, especially through the regulation of cell cycle and unscheduled proliferation.

Cellular proliferation and growth is regulated by E2F transcription factors through multiple downstream target genes, such as cyclins, c-myc, and so on. Several investigations reported that overexpression of E2F1 had clinical relevance in various types of cancers [35, 36]. E2F1 overexpression may promote cellular proliferation or cell cycle progression through upregulating the transcription of genes contributing to G1-S transition [37]. E2F4 was also reported to play important roles in the cell progression in various cancers. E2F4 takes part in the transcription regulation of multiple key genes in the Burkitt lymphoma tumorigenesis [38, 39]. In this study, the significant transcription factor targets included E2F2 and E2F4, which suggested that CDH23 may play crucial roles in cell progression of DLBCL via interacting with E2F2 and E2F4.

MiRNAs, which are critical for post-transcriptional regulation of gene expression, play important roles in carcinogenesis. In this study, we found the significant miRNA target of CDH23 was (TTCCGTT) MIR-191 in DLBCL. MiR-191, which was reported to serve as a tumor promoter in various tumors, was identified as an important oncogenic miRNA. MiR-191 could promote tumorigenicity in breast via interacting with estrogen and radiation survival in prostate cancer by interacting with Retinoid X receptor (RXRA) [40–42]. MiR-191 was also found to take important part in maintaining immune homeostasis, especially via supporting T-cell survival and modulating B cell development [43, 44]. These results indicated that CDH23 may play important roles in tumorigenesis, serve as a prognostic marker and regulate the microenvironment of DLBCL.

## CONCLUSIONS

The expression of CDH23 is reduced via DNA methylation significantly in DLBCL tissues. Reduction of CDH23 represents poor outcome of DLBCL patients. CDH23 may play important roles in immune cell infiltration of DLBCL. The enrichment functions of CDH23 show that CDH23 takes part in the essential biological functions, including cancer cell growth, cell metastasis, cell adhesion, cell cycle, drug catabolic process, leukocyte mediated immunity, DNA replication, DNA repair, and so on. CDH23 also associates with drug catabolic process. The significant kinase targets of CDH23 include CDK1 and CDK2, the significant transcription factor targets of CDH23 include E2F1 and E2F4, and the significant miRNA target of CDH23 is MIR-191 in DLBCL. All these results indicate that CDH23 may act as an essential role in DLBCL.

## MATERIALS AND METHODS

### The GEPIA analysis

GEPIA is a gene expression profiling and interactive analysis web server for cancer and normal samples. It is a web tool based on The Cancer Genome Atlas (TCGA) and Genotype-Tissue Expression (GTEx) data to deliver customizable functionalities. GEPIA provides important customizable and interactive analysis including profiling plotting, differential expression analysis, correlation analysis, similar gene detection, patient survival analysis, and dimensionality reduction analysis [45]. In this study the GEPIA was used to analyze the CDH23 expression level and prognostic value in DLBCL. And further the prognostic value of CDH23 related key genes was also analyzed.

### The expression analysis of CDH23 in DLBCL by the GEO datasets analysis

Gene expression profile datasets of the DLBCL and noncancerous tissues, GSE32018 and GSE56315, were obtained from NCBI-GEO (https://www.ncbi.nlm.nih.gov/geo/). For GSE32018, the GEO datasets platform was GPL6480 (Agilent-014850 Whole Human Genome Microarray 4x44K G4112F, Agilent Technologies, Santa Clara, CA, USA.) and for GSE56315 was GPL570 (Affymetrix Human Genome U133 Plus 2.0 Array, Affymetrix, Santa Clara, CA, USA.). There were 55 DLBCL tissues and 33 normal tonsil tissues in GSE56315 dataset. 22 DLBCL tissue samples, 7 normal lymph nodes samples and 6 normal tonsil tissues samples were profiled for the GSE32018 dataset. The expression level of CDH23 in DLBCL and normal tissues was obtained from GEO datasets and then analyzed.

Further we analyzed the CDH23 expression level in DLBCL cell lines with or without treating with demethylating agent decitabine for 48 hours from GSE27226, the GEO datasets platform was GPL6947 (Illumina HumanHT-12 V3.0 expression beadchip, Illumina, San Diego, CA, USA) [46].

### Genetic alterations of CDH23 in DLBCL

The cBioPortal database (http://cbioportal.org) is a useful Web resource, which provides exploring, visualizing, and analyzing multidimensional cancer genomics data [47, 48]. In this study we explored the aberration type, co-mutations and the mutated location of *CDH23* in DLBCL via cBioportal analysis.

### The LinkedOmics analysis

The LinkedOmics database (http://www.linkedomics.org) contains multi-omics data and clinical data for various cancers, including 32 cancer types, totally 11 158 patients from The Cancer Genome Atlas (TCGA) project. This database provides a unique platform that can be used for accessing, analyzing and comparing cancer multi-omics data within and across tumor types [49]. We used the “LinkFinder” module to identify the differentially expressed genes in the TCGA DLBCL cohort. The correlation of *CDH23* methylation value and expression level was further analyzed. The analysis of kinase targets, miRNA targets and so on for CDH23 was performed by the “LinkInterpreter” module. Results were analyzed for significance using the Pearson Correlation test. The p value cutoff was 0.05.

### The TIMER analysis

TIMER web server (https://cistrome.shinyapps.io/timer/), a comprehensive resource, can be employed for systematical investigating molecular characterization of tumor-immune interactions. TIMER provides 6 major analytic modules. The associations between immune infiltrates and a wide-spectrum of factors, including gene expression, somatic mutations, somatic copy number alterations and clinical outcomes can be interactively explored via TIMER. The TIMER algorithm estimates the abundance of six immunes infiltrates, including B cells, CD8+ T cells, CD4+ T cells, Macrophages, Neutrophils, and Dendritic cells [50]. The relation between the expression level of CDH23 and immune infiltration was investigated in this study via TIMER database.

### Protein-protein network construction via GeneMANIA

GeneMANIA (https://genemania.org/) is a widely used web server that can be usually used for predicting the function of selected genes and performing protein-protein interaction (PPI) network analysis [51]. Via this online tool, selected gene or gene lists can be analyzed by bioinformatic methods, including gene co-expression, physical interaction, gene co-location, gene enrichment analysis, and website prediction. The PPI network for CDH23 was constructed via GeneMANIA in this investigation.

### The DNA methylation interactive visualization database (DNMIVD) analysis

DNMIVD (http://www.unimd.org/dnmivd/) is a database that allows researchers to build molecular models for diagnosis and prognosis of cancer based on methylation of DNA, and visualizes the methylation profile of gene promoters and CpGs from various aspects [52–54]. We analyzed the expression level and methylation value of *CDH23* in breast invasive carcinoma (BRCA) and normal tissues, and further calculated the Pearson correlation between methylation of *CDH23* promoter and Fragments Per Kilobase of exon model per Million mapped fragments (FPKM) in BRCA.

### Statistical analysis

Independent t-test was employed to analyze the difference of CDH23 expression between tumor samples and normal samples. The relationship between the expression level of CDH23 and DLBCL patients prognosis was analyzed by Kaplan-Meier survival analysis and log-rank test via GEPIA database. Pearson correlation test was employed to explore the association between CDH23 expression and correlated genes expression. *P*-values <0.05 indicated significance in this study.

## Supplementary Material

Supplementary Figures

Supplementary Tables
