# Supplementary material for: Methylation silencing CDH23 is a poor prognostic marker in diffuse large B-cell lymphoma
Source: Aging (Albany NY). 2021 Jul 12;13(13):17768–88. doi: 10.18632/aging.203268 (PMC8312441; doi:10.18632/aging.203268)
Supplement: Supplementary Figures [file aging-13-203268-s001.pdf]

SUPPLEMENTARY FIGURES

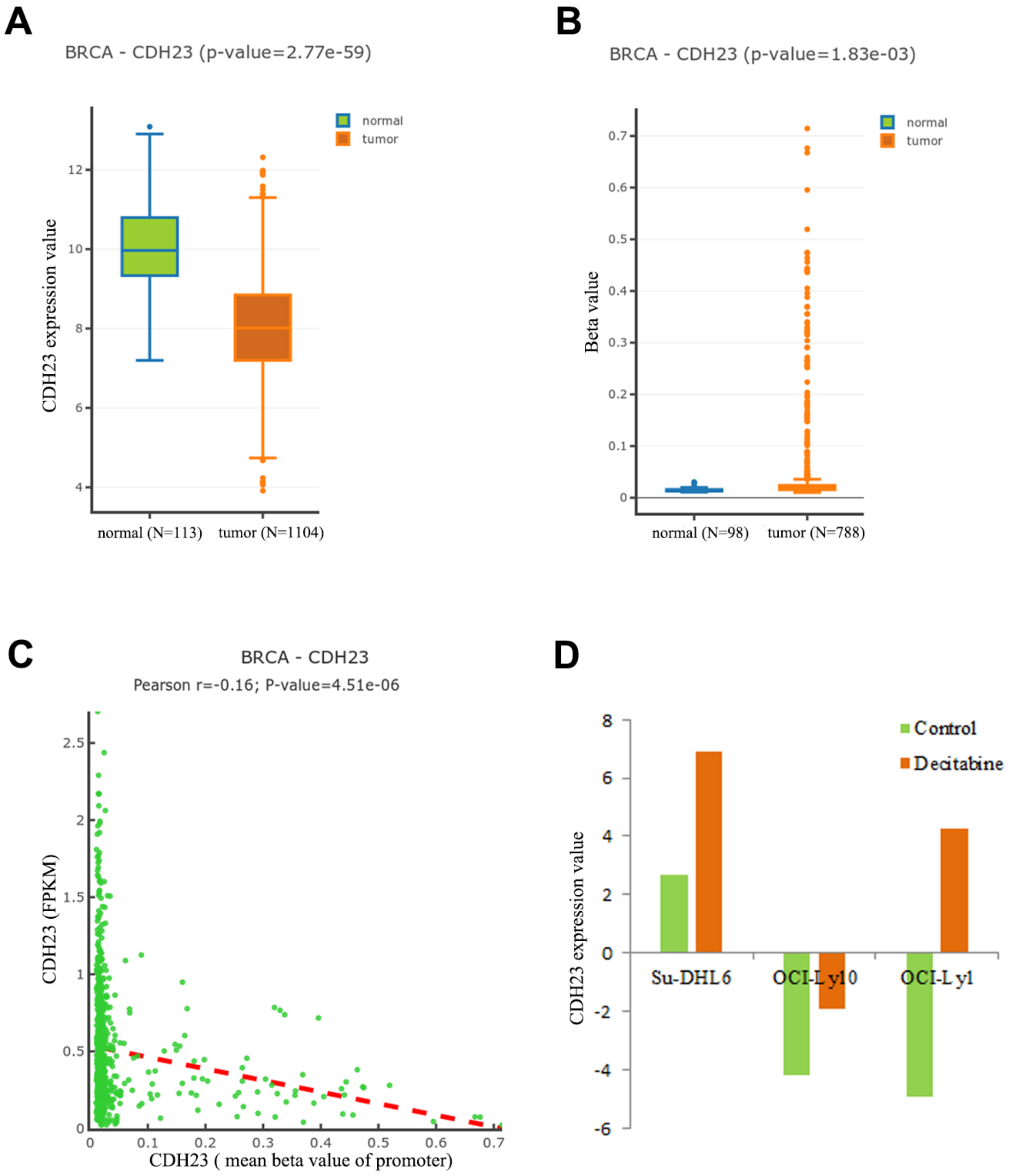

Supplementary Figure 1. The verification of epigenetic regulation of CDH23. (**A**) The expression level of CDH23 in BRCA tissues and normal tissues (DNMIVD). (**B**) The methylation value of *CDH23* in BRCA tissues and normal tissues (DNMIVD). (**C**) The correlation of CDH23 expression and promoter methylation value in BRCA (DNMIVD). (**D**) The expression level of CDH23 with or without the treatment of demethylating agent decitabine in DLBCL cell lines, including Su-DHL6, OCI-Ly10 and OCI-Ly1 (GEO).

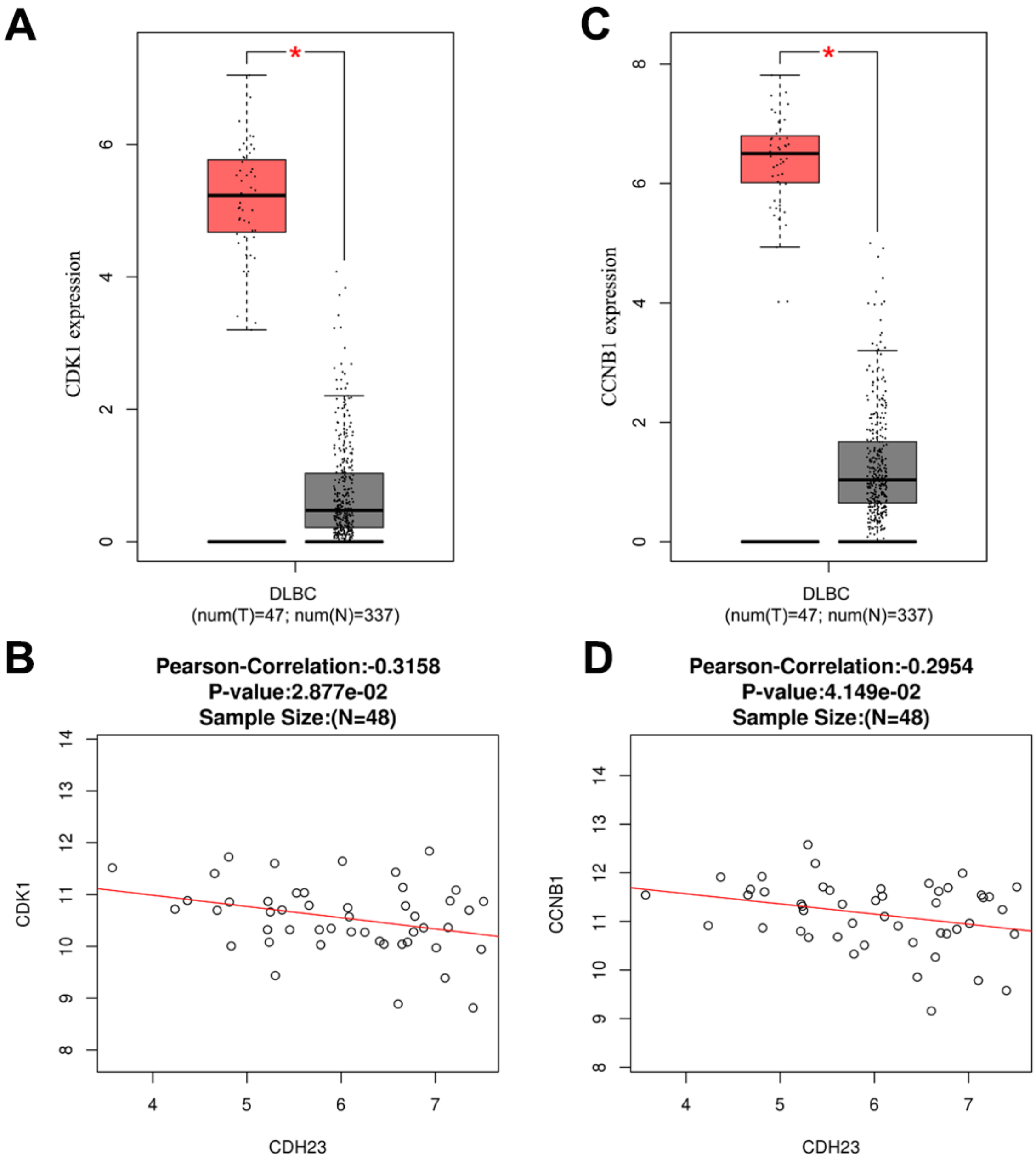

Supplementary Figure 2. The expression level of CDK1 and CCNB1, and the correlation between their expression level with CDH23 in DLBCL. (**A**) The expression level of CDK1 in DLBCL tissues and normal tissues (GEPIA). (**B**) The correlation of CDK1 expression level and CDH23 expression level (LinkedOmics). (**C**) The expression value of CCNB1 in DLBCL tissues and normal tissues (GEPIA). (**D**) The correlation of CCNB1 expression level and CDH23 expression level (LinkedOmics).
