# Supplementary material for: Methylation silencing CDH23 is a poor prognostic marker in diffuse large B-cell lymphoma
Source: Aging (Albany NY). 2021 Jul 12;13(13):17768–88. doi: 10.18632/aging.203268 (PMC8312441; doi:10.18632/aging.203268)
Supplement: Supplementary Tables [file aging-13-203268-s002.pdf]

SUPPLEMENTARY TABLES

Supplementary Table 1. Significantly enriched representative GO annotations (biological process) of CDH23 in DLBCL (LinkedOmics).

Description	Enrichment ratio	FDR	LeadingEdgeGene
**drug catabolic process**	1.95	5.91 E-04	ACHE;GPX1;PM20D2;CHIT1;IDUA;PIPOX;RENBP;SDSL;MPO;ALDH2;PRDX1;CTSH;MAOB;OXCT1;MAOA;SULT1A1;COMT;CYP1A2;SULT1A2;AMDHD2;GNPDA2;CYP2S1;PAOX;AMPD3;ALDH3B1;PCK2;NAGK;IL4I1;MT3;GLDC;PCBD1;GCSH;SMOX;DPYS;AMT;NNT;EPX;OVGP1;IDE;GPX3;NPL;CYP2F1;CYP2B6;COLQ;LPO;CYP4B1.
**DNA repair**	1.52	1.76 E-04	ACTR2;MSH2;NUDT16;PDS5B;DHX9;UCHL5;WDHD1;STUB1;CDK2;UBC;MSH3;SUPT16H;DTL;SIRT1;RAD54B;PMS1;ESCO2;FANCM;RAD21;SFPQ;SLC30A9;USP28;BRIP1;CDC5L;MCM8;CIB1;FOXM1;DEK;MAGEF1;PRMT6;ATXN3;NONO;LIG3;RMI1;ETAA1;USP43;POLD3;CHEK1;FANCC;PDS5A;NUCKS1;POLA1;RAD52;CDC7;MPG;RNF169;UBE2N;EXO1;PRKCG;KLHL15;BRCA1;CLSPN;FBXO6;EP300;PAXIP1;SMC1A;BARD1;NFRKB;DNA2;RNF138;MORF4L2;NIPBL;ASCC1;PIAS4;CUL4A;ERCC4;TREX1;USP1; etc.
**leukocyte mediated immunity**	1.40	1.10 E-04	IL18;MMP9;GSN;PLD1;VAMP3;NPC2;ATG7;PSAP;CLU;PYCARD;FTH1;CDA;VAT1;C8G;ACTR2;CD63;CHIT1;AGPAT2;MAN2B1;TOM1;CTSD;APAF1;PTGES2;BRI3;ITGAX;MSH2;GRN;LAMP1;DPP7;LTA4H;MMP25;VNN1;SERPINA3;C5AR1;CTSA;HEXB;CST3;NCR3;ATAD5;PYGB;GAA;PI4K2A;LTA;ZP3;ORM2;C1R;C1S;RAC1;SERPINB6;CFP;S100A13;ORM1;ATP6V0C;TCIRG1;CTSZ;ARSA;TNFRSF1B;ACLY;MVP;PKP1;MANBA;ASAH1;CD68;RAB24;TMEM173;MPO;C1RL;GM2A;TIMP2;FUCA2;TMEM63A;CHI3L1;PRDX1;CTSH;FTL;EMP2;ITGB2;LRRC7;HLA-F;PLEKHO2;GSTP1;PVR;PRKCZ;CHRNB4;CYFIP1;ITGAM;VAMP2;ANPEP;ANXA2;GALNS;RAB3A;RAB6A; etc.
**cell cycle**	1.36	9.54 E-09	CDK18;SNX33;GAS2L1;PIWIL4;CEBPA;KLHDC8B;OBSL1;FBXW5;AVPI1;UBD;TOP2A;CENPF;SPHK1;ACTR2;TOM1L2;LMNA;MEI1;CD2AP;RASSF4;NUPR1;TPX2;RNF2;ASPM;APAF1;ZNF385A;PRIM2;MSH2;NUDT16;KIF15;CAPN3;SMARCD3;CENPJ;CCNF;XPO1;OPTN;CDC73;KIF14;SASS6;SUZ12;KIF18B;VASH1;STEAP3;RRM1;CREBL2;PDS5B;NSL1;ATAD5;CIT;KIF20A;GPNMB;INTS7;RACGAP1;HNRNPU;ZNF207;RBM7;CKAP5;CEP57;NCAPD3;WDHD1;FAM83D;STIL;CTDSPL;PPME1;CDCA8;SMC4;SPAG5;CDK2;FBXL15;MKI67;LIN9;GPSM2;PPAT;CYP27B1;ZFP36L2;PPM1D;IPO7;TUBGCP4;KIF18A;CTCFL;DONSON;TTK;KIFC1;CABLES2;TPR;CASP8AP2;HJURP;MSH3;SMC2;ANLN;DHFR;CUL5;DTL;CCNE2;CDC23;E2F8;SIRT1; etc.

Abbreviations: FDR, false discovery rate from overrepresentation enrichment analysis (ORA).

Supplementary Table 2. Significantly enriched kinase-target networks of CDH23 in DLBCL (LinkedOmics).

GeneSet	Enrichment ratio	FDR	LeadingEdgeGene
**Kinase_CDK2**	1.48	0.009	SORBS3;VIM;CTTN;CENPF;TPX2;PDS5B;CDK2;MKI67;TPR;DTL;THRAP3;CDC23;CSNK2A1;ZMYM3;NPAT;HNRNPK;RBM27;CDC5L;GIGYF2;FOXM1;NCAPH;DPF2;CDC27;DLGAP5;RRN3;DLG1;CCNE1;NUP98;LIG3;MED1;CHEK1;TUBG1;USP37;FOXC1;NUCKS1;CDC6;PHF6;ZYX;CDC7;LMNB2;SNW1;DNMT1;LARP1;RBL1;BRCA1;NCL;C17orf49;EFHD2;SUDS3;ARHGAP19;BARD1;E2F3;MCM4;EGLN2;NOSIP;HNRNPUL1;NUP153;NUP107;ARID4A;POLL;CAMSAP1;APC;ZC3H11A;DPYSL3;UBXN1;POLH;ELAVL1;DIAPH3;UHRF1;ANAPC13;MDC1;SUPT6H;MTA1;ELK4;UBE2O;TOPBP1;TNKS1BP1;RBBP8;POLR2A;ANKRD17;NUFIP2;ANAPC1;SRRM1;ATF2
**Kinase_CDK1**	1.46	0.016	SQSTM1;VIM;CTTN;TOP2A;LMNA;TPX2;LMNB1;IRS2;XPO1;PDS5B;LBR;SPAG5;MKI67;TPR;DTL;CDC23;SIRT1;CSNK2A1;TOP1;PRDX1;RSF1;ZMYM3;NCOA3;HNRNPK;NUSAP1;CREB1;TMPO;KIF20B;GIGYF2;FOXM1;CDC27;DLGAP5;U2AF2;DLG1;NUP98;KIF11;SOD2;LIG3;CHEK1;NUCKS1;CEP55;NCAPG;CENPA;CDC7;LMNB2;NAGK;DNMT1;TEX14;FBXO43;BRCA1;NCL;EFHD2;ESPL1;BARD1;EIF4EBP1;HMGCS1;NEDD1;WEE1;USP1;CKAP2;GAPVD1;PHF8;CD3EAP;PRC1;LATS1;ZC3H11A;ELAVL1;UHRF1;CCNB1;CUEDC2;MDM4;POLR2A;EPB41;RPS6KB1;SLK;ANAPC1;PIK3C2A

Abbreviations: FDR, false discovery rate from overrepresentation enrichment analysis (ORA).

Supplementary Table 3. Significantly enriched miRNA-target networks of CDH23 in DLBCL (LinkedOmics).

GeneSet	Enrichment ratio	FDR	LeadingEdgeGene
**GGCGGCA,MIR-371**	2.93	0.97	PTGES2;POM121;DYNLL2
**TTCCGTT,MIR-191**	2.53	0.04	ATP2B2;PHC2;TMOD2;CEBPB;ZCCHC24;GAP43;TAF5;LIN54;AMMECR1;PLCD1;BRMS1L;OXSR1;MAPRE3;AMMECR1L;MAP3K12

Abbreviations: FDR, false discovery rate from overrepresentation enrichment analysis (ORA).

Supplementary Table 4. Significantly enriched representative transcription factor-target networks of CDH23 in DLBCL (LinkedOmics).

GeneSet	Enrichment ratio	FDR	LeadingEdgeGene
**V$E2F4DP1_01**	1.67	2.14E-04	EHBP1;MSH2;SASS6;SYNGR4;SLCO3A1;ATAD5;POLG;KCNS2;ATAD2;PPM1D;IPO7;CASP8AP2;E2F8;UBR7;ARHGAP11A;E2F7;TMPO;HNRNPA2B1;HMGXB4;CDC5L;MCM8;ZNF524;POLD3;FANCC;USP37; etc.
**V$E2F_02**	1.66	2.14E-04	EHBP1;MSH2;SASS6;SYNGR4;SLCO3A1;ATAD5;KCNS2;ATAD2;PPM1D;IPO7;CASP8AP2;E2F8;UBR7;ARHGAP11A;E2F7;TMPO;HNRNPA2B1;HMGXB4;CDC5L;MCM8;ZNF524;POLD3;FANCC;USP37;POLA1; etc.
**V$E2F1DP2_01**	1.66	2.14E-04	SLITRK4;EHBP1;MSH2;SASS6;SYNGR4;SLCO3A1;ATAD5;KCNS2;ATAD2;PPM1D;IPO7;CASP8AP2;E2F8;UBR7;ARHGAP11A;E2F7;TMPO;HNRNPA2B1;HMGXB4;CDC5L;MCM8;ZNF524;POLD3;FANCC;USP37;POLA1;TRA2B;GABRB3;CDC6;PPIG;TLE3;DNMT1; etc.
**V$E2F1_Q3**	1.64	3.34E-04	TRIM47;EPHB2;EHBP1;MSH2;KIF15;SASS6;SYNGR4;SLCO3A1;PDS5B;ATAD5;ATAD2;PPM1D;IPO7;CASP8AP2;E2F8;UBR7;ARHGAP11A;E2F7;TMPO;MCM8;SERBP1;CACNA1G;ZNF524;CNOT3;POLD3;USP37; etc.
**V$NFMUE1_Q6**	1.50	0.011	CDH23;RBM12;KIF15;NFYA;INTS7;ZNF207;CD4;RAC1;ATP6V0C;SMC4;ARNT;DHX15;TSKU;XPR1;FASTK;RBM19;SFPQ;VAMP2;HMGXB4;GIGYF2;SERBP1;CPSF2;CELF1;PRMT6;CCNE1;MED1;CNOT3;NKIRAS2;SLAIN1;TRA2B;SNAP25;STRN4;NSD1;UBXN11; etc.
**V$CREB_Q4**	1.45	0.018	MAP1LC3A;CD2AP;FAM19A1;CLSTN3;SIK2;INTS7;PQLC1;CEP57;WDHD1;FAM167A;ATP6V0C;RBP5;RELB;TMEM86A;ZMYND15;RAB24;HNRNPA2B1;NPTX1;RAB3A;RAB6A;NUP98;ARL4D;ASPHD1;ANK2; etc.

Abbreviations: FDR, false discovery rate from overrepresentation enrichment analysis (ORA). V$, the annotation found in Molecular Signatures Database (MSigDB) for transcription factors (TF).
